# Long Non-Coding RNA Malat1 Increases the Rescuing Effect of Quercetin on TNFα-Impaired Bone Marrow Stem Cell Osteogenesis and Ovariectomy-Induced Osteoporosis

**DOI:** 10.3390/ijms24065965

**Published:** 2023-03-22

**Authors:** Lu Feng, Zhengmeng Yang, Nan Hou, Ming Wang, Xuan Lu, Yucong Li, Haixing Wang, Yaofeng Wang, Shanshan Bai, Xiaoting Zhang, Yuejun Lin, Xu Yan, Sien Lin, Micky D. Tortorella, Gang Li

**Affiliations:** 1 Centre for Regenerative Medicine and Health, Hong Kong Institute of Science & Innovation, Chinese Academy of Sciences, Hong Kong SAR, China; 2 Stem Cells and Regenerative Medicine Laboratory, Li Ka Shing Institute of Health Sciences, The Chinese University of Hong Kong, Prince of Wales Hospital, Shatin, Hong Kong SAR, China; 3 Musculoskeletal Research Laboratory, Department of Orthopaedics & Traumatology, Faculty of Medicine, The Chinese University of Hong Kong, Prince of Wales Hospital, Shatin, Hong Kong SAR, China; 4 The CUHK-ACC Space Medicine Centre on Health Maintenance of Musculoskeletal System, The Chinese University of Hong Kong Shenzhen Research Institute, Shenzhen 518000, China

**Keywords:** Malat1, osteoporosis, quercetin, BMSCs, osteogenesis

## Abstract

Osteoporosis, a common systematic bone homeostasis disorder related disease, still urgently needs innovative treatment methods. Several natural small molecules were found to be effective therapeutics in osteoporosis. In the present study, quercetin was screened out from a library of natural small molecular compounds by a dual luciferase reporter system. Quercetin was found to upregulate Wnt/β-catenin while inhibiting NF-κB signaling activities, and thereby rescuing osteoporosis-induced tumor necrosis factor alpha (TNFα) impaired BMSCs osteogenesis. Furthermore, a putative functional lncRNA, Malat1, was shown to be a key mediator in quercetin regulated signaling activities and TNFα-impaired BMSCs osteogenesis, as mentioned above. In an ovariectomy (OVX)-induced osteoporosis mouse model, quercetin administration could significantly rescue OVX-induced bone loss and structure deterioration. Serum levels of Malat1 were also obviously rescued in the OVX model after quercetin treatment. In conclusion, our study demonstrated that quercetin could rescue TNFα-impaired BMSCs osteogenesis in vitro and osteoporosis-induced bone loss in vivo, in a Malat1-dependent manner, suggesting that quercetin may serve as a therapeutic candidate for osteoporosis treatment.

## 1. Introduction

Osteoporosis is a chronic bone disease caused by a systemic disorder in bone homeostasis, resulting in loss of bone mass and microarchitecture deterioration. Osteoporosis is prevalent in postmenopausal women, which may increase fracture risk and result in economic and clinical burden for families and society [1]. As a degenerative disease, osteoporosis is highly age-corelated, caused by excessive bone resorption mediated by osteoclasts and insufficient bone formation by osteoblast intervention [2].

Bone homeostasis is dynamically modulated by bone formation and resorption, which are controlled by a variety of osteogenic and osteoclastogenic regulators of the immune, neuronal, and musculoskeletal systems [3]. With the progression of osteoporosis, some cytokines are released into the bone microenvironment, which may further upregulate bone resorption and inhibit bone synthesis through certain functional signaling pathways. As shown in previous studies, circulating monocytes directly participate in osteoclastogenesis by providing precursors and secreting osteoclastogenic factors such as IL-1, IL-6 and tumor necrosis factor alpha (TNFα) [4]. TNFα has been shown to play an important role in estrogen deficiency-mediated osteoporosis. As a prevalent pro-inflammatory cytokine, TNFα promotes osteoclast formation by promoting RANKL-induced osteoclastogenesis. Specifically, RANKL, activated by TNFα, triggers phosphorylation of IκBα as well as the release of p65/p50 heterodimer and nucleus translocation, thereby activating NF-κB signaling and upregulating the expression of osteoclast genes, such as c-Fos and NFATc1 [5]. The presence of TNFα also downregulates new bone formation by inhibiting the viability and differentiability of bone marrow mesenchymal stem cells (BMSCs), thereby reducing osteogenesis and suppressing osteogenic marker expression, including Runx2 and Osterix [6]. TNFα may inhibit the osteogenic activity of BMSCs in a Wnt/β-catenin signaling-dependent manner. As demonstrated in previous studies, Wnt/β-catenin signaling plays a critical role in skeletal development by inducing BMSCs to differentiate into mature osteoblasts [7]. In the inflammation condition, the high environmental TNFα-induced NF-κB activation could also negatively regulate Wnt/β-catenin signaling, and thereby inhibit osteogenesis due to their interacting mechanism [8].

In previous studies, several natural small molecules were found to be effective therapeutics in osteoporosis by modulating Wnt/β-catenin and NF-κB signaling activities. Our previous studies indicated that sesamin, a natural small molecule, plays a therapeutic role in osteoporosis by promoting Wnt/β-catenin and inhibiting NF-κB signaling pathways. The in vivo administration of sesamin could significantly ameliorate osteoporosis-induced bone loss [9]. While another pentacyclic triterpenoid, Asiatic acid, could inhibit osteoclast differentiation and alleviate postmenopausal osteoporosis by modulating TGFβ and NF-κB signaling [10]. Quercetin is a plant flavonoid compound widely distributed in leaves and seeds of fruits and vegetables. As a common dietary ingredient, it shows several beneficial effects including antioxidant, antitumoral, antimicrobial anti-inflammation and antiviral functions. In a previous study, quercetin was proved a potential therapeutic candidate for alleviating ovariectomy-induced osteoporosis [11]. Quercetin administration promoted the proliferation and differentiation of BMSCs and increased the number of osteoblasts, thereby increasing bone mass and structures [12]. However, the precise regulating mechanism of quercetin on osteoporosis has not been fully uncovered yet.

LncRNAs have been shown to act as key regulators in osteoporosis. Based on previous studies, several ubiquitous lncRNAs, including Malat1, H19, Hotair, MEG3 and Dancr, were previously reported to be associated with osteoporosis. Particularly, they were also reported to be involved in Wnt/β-catenin and NF-κB signaling activities [13]. In this study, we have used a drug screening system to identify the modulating effects of quercetin on both Wnt/β-catenin and NF-κB signaling pathways. We also demonstrated its in vitro rescuing effect on TNFα-impaired BMSCs osteogenic activities as well as the in vivo therapeutic role of quercetin on an osteoporotic mouse model. The lncRNA Malat1 was shown to be the key modulating factor in the regulating mechanism of quercetin on in vitro BMSCs osteogenesis and in vivo osteoporosis development.

## 2. Result

### 2.1. Quercetin Could Upregulate Wnt/β-Catenin and Downregulate NF-κB Signaling

**Figure 1:**
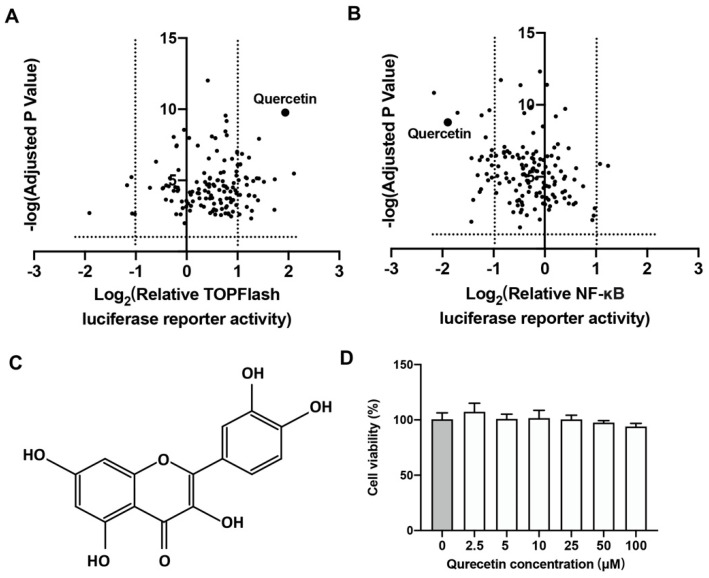
Screening of small molecules repressing the luciferase activity of TOPFlash and NF-κB response element system A&B. Volcano plot analysis of TOPFlash (**A**) and NF-κB response element (**B**) luciferase activities upon small molecule library treatment. Quercetin was highlighted. Molecular conformation of quercetin (**C**). Cytotoxicity assay of mouse BMSCs after treated with quercetin at different concentrations for 3 days (*n* = 6) (**D**).

A cell-based luciferase activity assay was applied to assess the promoter activities of natural small molecules on both Wnt/β-catenin and NF-κB signaling by using TOPFlash and NF-κB RE luciferase reporter systems, respectively. The volcano plot indicated that a herb-derived bioflavonoid, quercetin, could activate Wnt/β-catenin signaling, while repressing NF-κB signaling with a relatively high significances (Figure 1A,B). The molecular conformation of quercetin showed that quercetin has a classic hydroxyflavone structure with five hydroxy groups placed at the 3-, 3′-, 4′-, 5- and 7-positions, as displayed in Figure 1C. The cell viability assay result showed that quercetin had no obvious toxic effect on BMSCs in the concentration range of 0 to 100 μM (Figure 1D).

### 2.2. Quercetin Rescued TNFα-Impaired BMSCs Osteogenesis via Increasing Wnt/β-Catenin and Decreasing NF-κB Signaling

**Figure 2:**
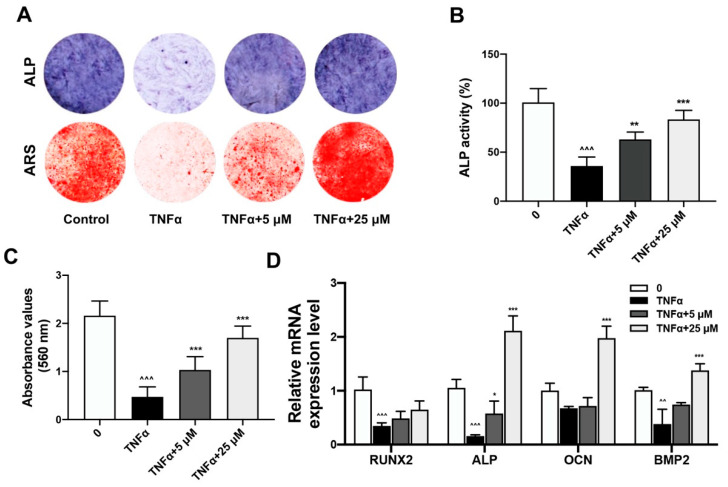
Quercetin rescued TNFα-impaired BMSCs osteogenesis. (**A**) The ALP staining at day 7 and the ARS staining of calcified nodules at day 14 after osteo-induction was performed. (**B**,**C**) Quantitative analysis of ALP activities (**B**) and ARS staining intensities (**C**). (**D**) The mRNA expression level of osteogenesis markers, including Runx2, Alp, Ocn and Bmp, were measured by real-time PCR after treatment with quercetin after osteo-induction for 7 days (*n* = 6; ^^^^ *p* < 0.01, ^^^^^ *p* < 0.001 versus the Control group; * *p* < 0.05, ** *p* < 0.01, *** *p* < 0.001 versus the TNFα treatment group).

The restoration of TNFα-impaired osteogenesis by quercetin was demonstrated by ALP and ARS assays. After pre-treatment with TNFα, the BMSCs osteogenesis was induced with osteo-induction medium together with the treatment of quercetin in a concentration gradient. Results of ALP staining at day 7 and ARS staining of calcified nodules at day 14 during osteo-induction revealed that TNFα administration significantly decreased the formation of the purple-colored insoluble precipitate catalyzed by alkaline phosphatase, which indicated impaired ALP activity. The ARS-stained, red-colored calcified nodules formed during BMSCs osteogenesis were also reduced upon TNFα treatment. The quercetin treatment could protect BMSCs osteogenic activity from TNFα impairment in a dose-dependent manner (Figure 2A). Quantitative analysis of the ALP activity and ARS staining intensity also indicated that the inhibitory effect of TNFα on BMSCs osteogenic activity was restored with an increased quercetin concentration (Figure 2B,C). Real-time PCR results also revealed the rescued mRNA expression of osteogenic markers, including Runx2, Alp, Ocn and Bmp, upon quercetin treatment, which demonstrated the protective effect of quercetin on TNFα-impaired BMSCs osteogenesis (Figure 2D).

**Figure 3:**
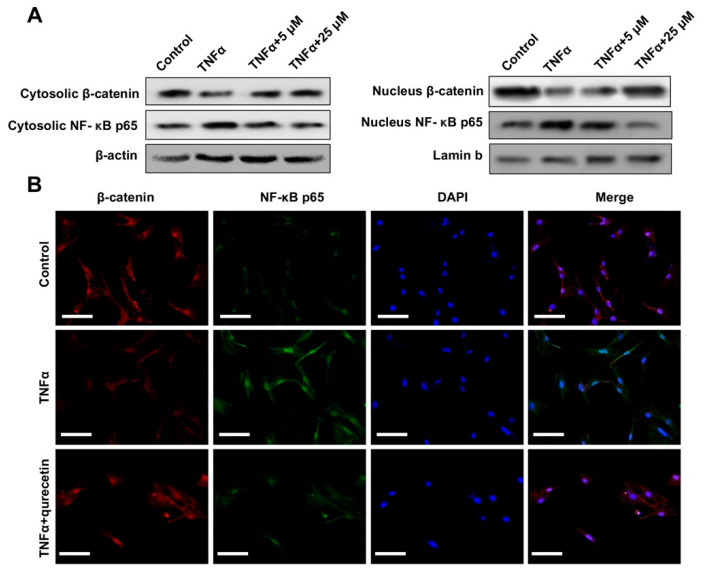
Quercetin reversed TNFα increased NF-κB and decreased Wnt/β-catenin signaling activities. (**A**) Western blot analysis of NF-κB p65 and β-catenin nucleus translocation of the BMSCs upon TNFα pre-treatment and quercetin administration. (**B**) Immunofluorescence (IF) staining analysis of the β-catenin and NF-κB p65 nucleus translocation of the BMSCs upon TNFα pre-treatment and quercetin administration. DAPI was included as an internal control. Scale bar: 100 μM.

The regulating effects of quercetin on Wnt/β-catenin and NF-κB signaling were further analyzed. The NF-κB p65 and β-catenin nucleus translocation in BMSCs pretreated with TNFα and administrated with quercetin were analyzed by Western blotting (Figure 3A, Supplementary Figure S1). TNFα pre-treatment could repress Wnt/β-catenin and activate NF-κB signaling activities in BMSCs by decreasing nucleus β-catenin while increasing NF-κB p65 nucleus translocation. Administration of 25 µM quercetin subsequently promoted nucleus β-catenin while inhibiting cytosolic and nucleus NF-κB p65 levels in a dose-dependent manner. Furthermore, immunofluorescence (IF) staining analysis also demonstrated the rescuing role of quercetin on β-catenin and NF-κB p65 nucleus translocation of the BMSCs impaired by TNFα (Figure 3B).

### 2.3. Quercetin Could Reverse the Expression of lncRNA Malat1 Downregulated by TNFα Treatment

**Figure 4:**
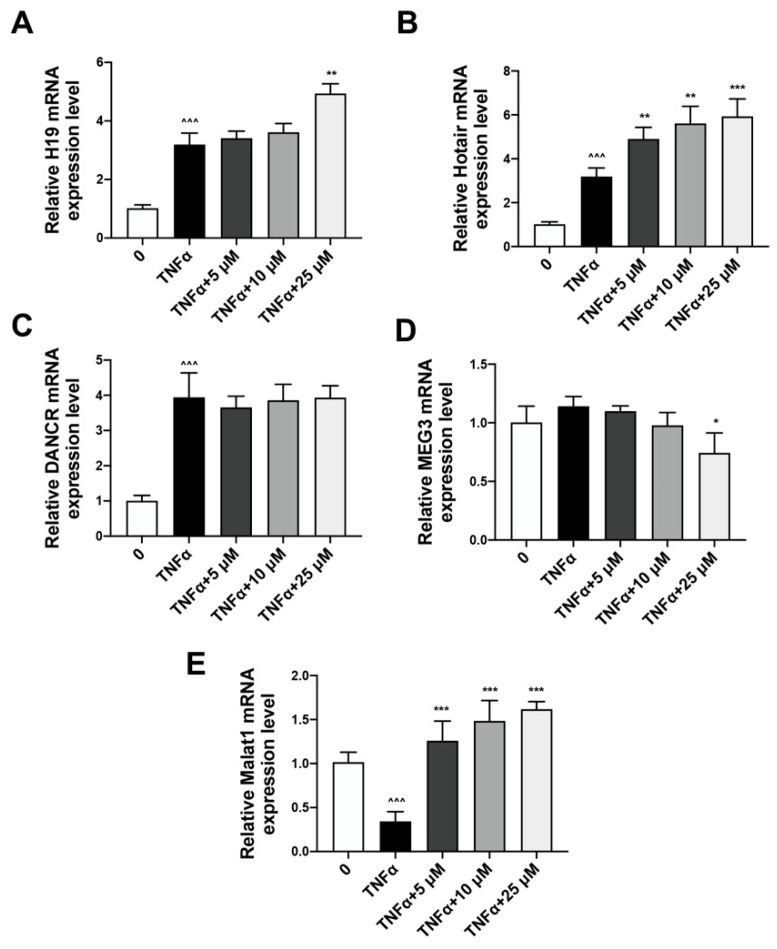
Identification of Malat1 as a quercetin-targeting gene in rescuing TNFα-impaired BMSCs osteogenesis A-E. The expression level of osteoporosis-related lncRNAs in BMSCs after being pre-conditioned with TNFα and treated with quercetin were detected by real-time PCR, including H19 (**A**), Hotair (**B**), Dancr (**C**), MEG3 (**D**) and Malat1 (**E**) (*n* = 6; ^^^^^ *p* < 0.001, * *p* < 0.05, ** *p* < 0.01, *** *p* < 0.001).

Real-time PCR analysis result indicated that the mRNA expression levels of H19, Hotair and Dancr were increased upon TNFα administration, and quercetin had a limited effect on them (Figure 4A–C). The mRNA expression level of lncRNA MEG3 was not obviously altered in response to TNFα and quercetin (Figure 4D). However, the relative Malat1 mRNA expression level was inhibited by TNFα while restored by a range of concentrations of quercetin (Figure 4E). Therefore, Malat1 may play an intermediary role in quercetin-enhanced BMSCs osteogenesis.

### 2.4. Knockdown of Malat1 Ameliorated the Rescuing Effect of Quercetin on TNFα-Impaired BMSCs Osteogenesis

**Figure 5:**
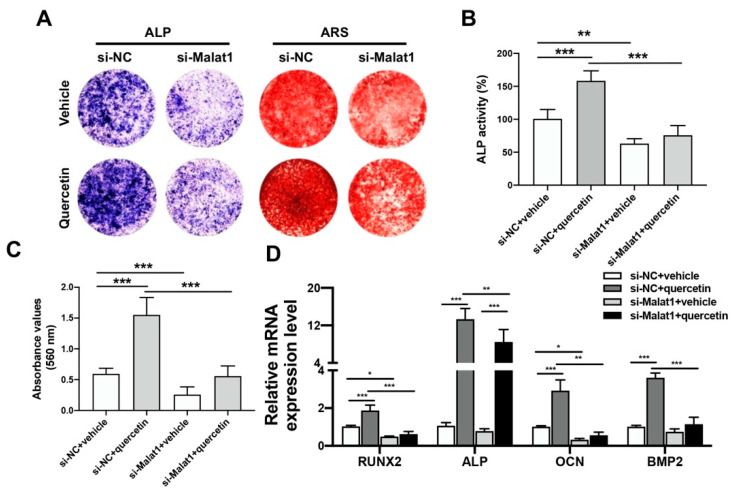
Malat1 mediated the rescuing effect of quercetin on TNFα-impaired BMSCs osteogenesis. (**A**) The BMSCs were Malat1 knockdown, pre-conditioned with TNFα for 12 h and treated with quercetin during osteo-induction for 7 days. ALP activity and calcified nodules formation were examined by ALP activity assay and ARS staining, respectively. (**B**,**C**) Quantitative analysis of ALP activities (**B**) and ARS staining intensities (**C**). (**D**) The mRNA expression level of osteogenesis markers, including Runx2, Alp, Ocn and Bmp, were measured by real-time PCR (*n* = 6; * *p* < 0.05, ** *p* < 0.01, *** *p* < 0.001).

The regulating role of Malat1 in the rescue of TNFα-injured BMSCs by quercetin was further demonstrated by siRNA-mediated Malat1 knockdown. BMSCs were transfected with Malat1 siRNA, preconditioned with TNFα and treated with 25 µM quercetin during osteo-induction. The Malat1 knockdown could attenuate the positive effect of quercetin on BMSCs osteogenic activity, as indicated by ALP and ARS staining results (Figure 5A). Quantitative analyzing of ALP activity and calcified nodules formation further revealed that quercetin could significantly upregulate osteogenic activity only in the si-NC group. Instead, the silencing of Malat1 significantly downregulated the overall osteogenic activity, regardless of quercetin administration (Figure 5B,C). The mRNA expression levels of osteogenic markers also showed that the administration of quercetin significantly upregulated the transcriptions of Runx2, Alp, Ocn and Bmp compared with the vehicle group. However, upon Malat1 knockdown, the promoting effect of quercetin on Runx2, Ocn and Bmp2 expression was only negligible (Figure 5D). Compared with the si-NC group, si-Malat1 transfection significantly decreased ALP activity, calcium nodule formation and osteogenesis marker expression of BMSCs. Malat1 silencing also attenuated the osteogenesis-promoting effect of quercetin. These data further demonstrated Malat1 as the key modulator in quercetin-induced osteogenesis.

**Figure 6:**
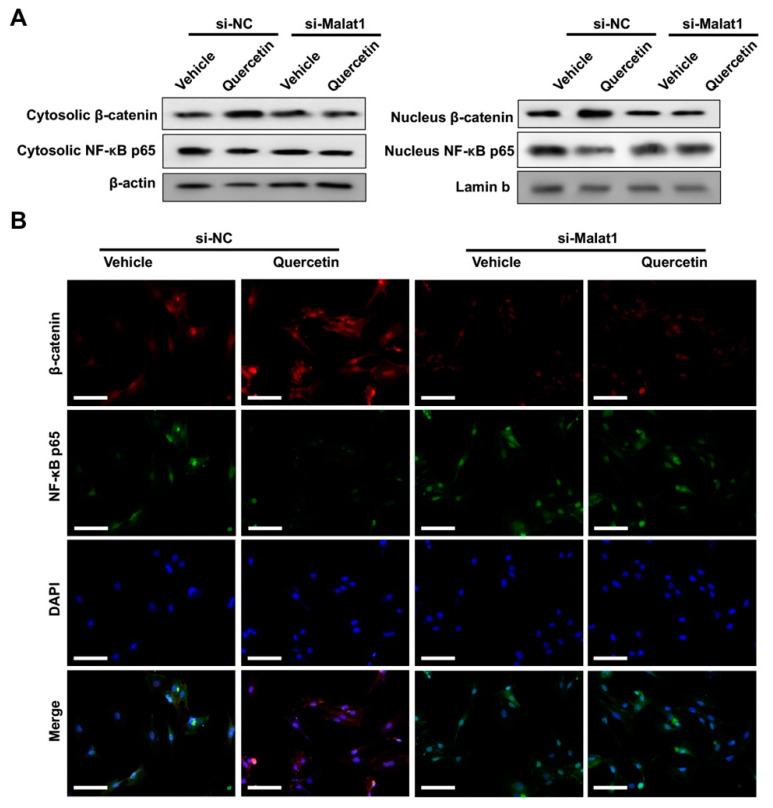
Effect of quercetin on promoting Wnt/β-catenin and repressing NF-κB signaling was ameliorated by Malat1 knockdown. The BMSCs were Malat1 knockdown, pre-conditioned with TNFα for 12 h and treated with quercetin for 3 days. (**A**) Western blot analysis of NF-κB p65 and β-catenin nucleus translocation of the BMSCs upon TNFα pre-treatment and quercetin administration. (**B**) Immunofluorescence (IF) staining analysis of the β-catenin and NF-κB p65 nucleus translocation of the BMSCs upon TNFα pre-treatment and quercetin administration. DAPI was included as an internal control. Scale bar: 100 μM.

Malat1 was also involved in the regulating effect of quercetin on Wnt/β-catenin and NF-κB signaling. Western blot analysis results showed that in the presence of Malat1, quercetin promoted β-catenin expression, while it inhibited NF-κB p65 translocation. However, upon Malat1 knockdown, the β-catenin expression was decreased while NF-κB p65 translocation was increased in BMSCs, regardless of quercetin administration (Figure 6A, Supplementary Figure S1). Immunofluorescence (IF) staining analysis of BMSCs of β-catenin or NF-κB p65 nucleus translocation after TNFα treatment and quercetin administration also indicated that quercetin acts in a Malat1-dependent manner (Figure 6B).

### 2.5. Quercetin Attenuates Bone Loss in the OVX Mouse Osteoporosis Model via Upregulating Malat1 Expression

**Figure 7:**
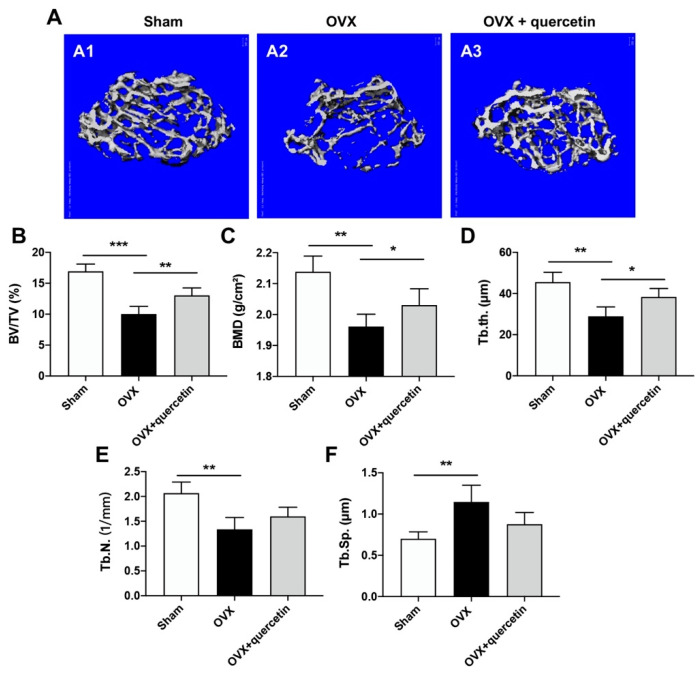
Microstructure properties of the trabecular bone of OVX mice after quercetin treatment. (**A**) Three-dimensional reconstruction of trabecular bone in the distal metaphysis of OVX mice after quercetin treatment. (**B**–**F**) Microstructure parameters, including bone volume/tissue volume (BV/TV) (**B**), bone mineral density (BMD) (**C**), trabecular thickness (Tb.Th) (**D**), trabecular number (Tb.N) (**E**) and trabecular space (Tb.Sp) (**F**) of the trabecular bone. Data are shown as mean ± SD (*n* = 6; * *p* < 0.05, ** *p* < 0.01, *** *p* < 0.001).

The therapeutic effect of quercetin on the osteoporosis was studied in an OVX-induced osteoporosis mouse model. OVX surgery induced bone loss and bone structure deterioration. Administration of quercetin significantly ameliorated osteoporosis progression compared with the OVX group, as shown by the 3D construction of trabecular bone in the distal metaphysis of OVX mice (Figure 7A). The microstructure parameters of trabecular bone, including bone volume/tissue volume (BV/TV), bone mineral density (BMD), trabecular thickness (Tb.Th.), trabecular number (Tb.N.) and trabecular space (Tb.Sp.), were analyzed. OVX could impair the microstructure of trabecular bone in femur metaphysis, as revealed by down-regulated BV/TV, BMD, Tb.Th., and Tb.N., and upregulated Tb.Sp. with statistic significance compared with the Sham group. However, all the parameters involved were reversely regulated upon quercetin administration (Figure 7B–F). The results indicate that quercetin significantly rescues bone mass loss induced by OVX.

**Figure 8:**
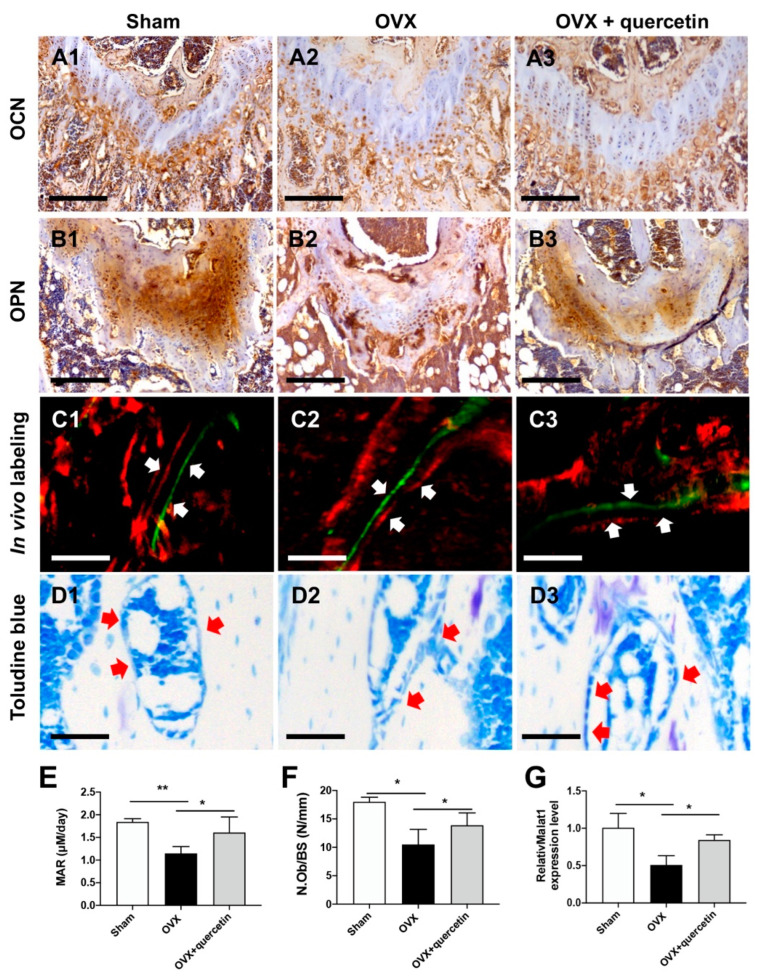
Histomorphometry of the distal femur from OVX mice upon quercetin treatment. (**A**,**B**) Representative images of femoral metaphysis with IHC staining using osteocalcin (OCN) (**A**) and osteopontin (OPN) (**B**) antibodies. Scale bar: 200 μM. (**C**,**D**) In vivo double labels (**C**) and Toluidine blue staining (**D**) of the distal femur sections. Red arrow: osteoblast. Scale bar: 100 μM. (**E**,**F**) Histomorphometric analysis of distal femur sections, including mineral apposition rate (MAR) (**E**) and number of osteoblasts per bone surface (N.Ob/BS) (**F**). (**G**) Serum level of Malat1 was revealed in OVX mice upon quercetin treatment. Data are shown as mean ± SD (*n* = 6; * *p* < 0.05, ** *p* < 0.01).

The histological and histomorphometrical analysis of the distal femur metaphysis was also performed. The IHC staining of the femoral metaphysis revealed that OVX severely inhibited the expression of osteogenesis markers, including osteocalcin (OCN) and osteopontin (OPN), while quercetin exhibited a counterbalanced function on the OVX impaired osteogenesis (Figure 8A,B). The in vivo double-labeling analysis revealed an accelerated bone growth rate following quercetin administration. Toluidine blue staining of the distal femur sections also showed that the impaired bone mass and reduced osteoblast number (red arrow) in the OVX group was partially rescued by quercetin (Figure 8C,D). Quantitative analysis of bone parameters showed a reduced osteogenesis rate (mineral apposition rate, MAR) and a decreased number of osteoblast/bone surface (N.Ob/BS) in the OVX group; these parameters were obviously restored by quercetin treatment (Figure 8E,F). In addition, serum Malat1 levels were decreased after OVX, and quercetin treatment could upregulate serum Malat1 levels to some extent (Figure 8G).

## 3. Discussion

In the present study, quercetin was found to rescue the TNFα-impaired BMSCs osteogenesis by promoting Wnt/β-catenin while inhibiting NF-κB signaling activities. Further investigation revealed that Malat1 was involved in the regulating mechanism of quercetin on BMSCs osteogenesis and the mouse osteoporosis process. Malat1 knockdown could abolish the stimulatory effect of quercetin on Wnt/β-catenin and its repressing effect on NF-κB signaling activities. Therefore, our study revealed the novel regulating mechanism of quercetin on osteogenesis and its therapeutic potential in the treatment of osteoporosis.

For osteoporosis treatment, several categories of drugs have been employed, aiming to promote bone regeneration or inhibit bone resorption. So far, antiresorptive agents, such as bisphosphonate and denosumab; anabolic agents, such as teriparatide and abaloparatide; and dual-acting agents such as romosozumab, have been approved for clinical use [14]. However, the current clinical drugs still exhibit some side effects and disadvantages. Anti-resorptive drugs have the shortcomings of a slow onset of action, a small effect size and poor bone structure reconstruction ability. Anabolic drugs need to be administrated daily and they may also increase the risk of osteosarcoma [15]. Dual-acting drug romosozumab could promote osteogenesis in a short time window period, but it may increase the risk of cardiovascular failure. Current studies revealed that some therapeutic compounds derived from nature plants have been discovered to have potential therapeutic effects in osteoporosis treatment with minimal side effects [16]. Umbelliferon, for instance, the derivative of coumarin, could facilitate bone formation via the Wnt/β-catenin signaling pathway [17].

As a natural molecule, quercetin has extraordinary properties in safety as an active ingredient of daily-used health products. In previous studies, quercetin was found to upregulate the BMSCs osteoblast differentiation [12]. However, the modulating mechanism by which quercetin rescues bone loss has not been systematically investigated. Previous studies indicated that the chronical postmenopausal osteoporosis may cause an inflammation environment in the patient’s bodies, with the signature of highly expression pattern the pro-inflammatory cytokines, including TNFα. BMSCs were stimulated by TNFα in an NF-κB-dependent manner [18]. Activated NF-κB could inhibit osteogenic differentiation of BMSCs by promoting β-catenin degradation through induction of the E3 ubiquitin protein ligases Smurf1 and Smurf2 [19]. On the other hand, the increased TNFα may also up-regulate DKK-1 and SOST, which directly inhibits the Wnt/β-catenin signaling pathway, and downregulates the mRNA expression of osteogenesis-related genes [20]. Specifically, Wang et al.’s study revealed that TNFα could induce NF-κB activation and further inhibit BMSCs osteogenesis by deactivating β-catenin [21]. The study further strengthened our hypothesis that the restoration of BMSCs osteogenesis activity and bone mass in the inflammation environment of osteoporosis conditions mainly depends on the dual regulation of both Wnt/β-catenin and NF-κB signaling activities. In our previous study, we found that sesamin, a natural small molecule, plays a therapeutic role in osteoporosis by promoting Wnt/β-catenin and inhibiting NF-κB signaling pathways. The in vivo administration of sesamin significantly ameliorated OVX bone loss, which sheds light on the current study to characterize novel small molecules as dual-signaling modulator in osteoporosis therapeutic study. In this study, we used the dual-luciferase assay system to screen out quercetin as an activator of Wnt/β-catenin and repressor of NF-κB signaling. Quercetin was further discovered to ameliorate TNFα-impaired BMSCs osteogenesis via reversing the abovementioned two signaling activities, which makes it an ideal therapeutic approach for osteogenesis-induced bone loss.

In our study, we also identified Malat1, a long non-coding RNA (lncRNA), as the mediator of the rescuing effect of quercetin on TNFα-impaired BMSCs osteogenesis. LncRNAs are RNAs approximately 200 nucleotides in length that do not translate proteins but play important roles in transcriptional and post transcriptional regulation. LncRNAs have emerged as important regulators of osteogenesis processes via different regulating mechanisms [22]. Several lncRNAs, including Malat1, H19, HOTAIR, MEG3 and DANCR, were largely involved in the osteoporosis. Malat1 and H19 could promote osteogenesis, while HOTAIR, MEG3 and DANCR inhibited osteogenesis in a Wnt/β-catenin-dependent manner [9,23,24,25,26]. Furthermore, MALAT1 repressed the LPS-induced inflammatory response by interacting with NF-κB [27]. In this study, the expression of H19, Hotair and Dancr was upregulated upon TNFα induction, but was not significantly reversed by quercetin administration. The expression of MEG3 exhibited negligible fluctuations with TNFα and quercetin treatment. However, the expression of Malat1 in BMSCs was inhibited by TNFα but effectively rescued by quercetin, which makes Malat1 a putative mediator of the quercetin’s treatment effect on TNFα-impaired osteogenesis. In our study, Malat1 knockdown could obviously diminish the modulating effect of quercetin on both Wnt/β-catenin and NF-κB signaling activities, as well as its promoting effect on BMSCs osteogenesis. Our study demonstrates the uniqueness and effectiveness of lnc Malat1 in quercetin ameliorated bone mass restoration.

In our study, OVX surgery enhanced trabecular bone resorption while inhibiting bone formation, causing bone microstructure deterioration in a mouse model. In addition, the expression of osteogenic markers, including OCN and OPN, regarding the viability and differentiation of osteoblasts, were decreased in the OVX group. Quercetin administration could effectively restore OVX-induced bone loss, which is consistent with previous research [28]. Specifically, the serum level of Malat1 was downregulated by OVX surgery, while quercetin treatment could obviously reverse it. In the clinical study, plasma level of MALAT1 is negatively correlated with the disease severity of postmenopausal osteoporosis, which suggested MALAT1 as a promising indicator of osteoporosis [29]. Our study demonstrated quercetin as an effective treatment for osteoporosis via regulating circulating Malat1expression level. However, our study still has some limitations. We have characterized the regulating role of quercetin on the Wnt/β-catenin and NF-κB signaling. Malat1 was also identified as the universal modulator of quercetin’s treatment effect on osteoporosis. However, we have not identified the direct binding protein target of quercetin. In silico molecule docking and drug-target association studies will be further performed to better understand the regulating mechanism of quercetin on BMSCs osteogenesis and osteoporosis.

In summary, our results indicated the in vitro regulating mechanism of quercetin on TNFα-impaired osteoporosis and in vivo treatment efficacy of quercetin on osteoporosis. The key mediating role of Malat1 in quercetin’s effect on osteoporosis has also been covered. Quercetin, as a natural molecular product, possesses a significant therapeutical effect on osteoporosis and fewer adverse reactions, which makes it an ideal drug candidate for rebalancing bone metabolism and treating osteoporosis, especially for the postmenopausal osteoporosis patients with a low serum MALAT1 level.

## 4. Material and Methods

### 4.1. Herb-Derived Small Molecule Library

An herb-derived natural small molecule library which contains 143 compounds was obtained from Selleck Chemicals (Harris Country, TX, USA). All these compounds are found in various medicinal plants. These compounds are dissolved in DMSO at 10 mM concentrations and stored at −180 °C.

### 4.2. Transient Cell Transfection and Dual-Luciferase Reporter Assay

Wnt/β-catenin response element (RE) luciferase reporter vector TOPFlash, NF-κB response element (RE) luciferase reporter vector and Renilla luciferase control reporter vectors were purchased from Upstate Cell Signaling (Charlottesville, VA, USA). The Malat1 small interfere RNAs (si-Malat1) and control siRNAs (si-NC) were synthesized by GenePharma (Shanghai, China). The human embryonic kidney 293 (HEK293) cells were purchased from ATCC (CRL-1573). BMSCs were isolated from the previous studies and stocked in our laboratory. The transient transfection was performed using Lipofectamine 3000 (Invitrogen, Waltham, MA, USA). To perform the high-throughput luciferase screening, the HEK293 cells were seeded in 96-well plates at the density of 5000 cells/well. After 80% confluency, the cells were co-transfected with TOPFlash or NF-κB RE reporter vector, together with Renilla vector, respectively. The cells were then treated with chemicals from library as mentioned before at a concentration of 10 μM for 24 h. The dual-luciferase assay was then conducted in a PerkinElmer VictorTM X2 2030 multilateral reader (Waltham, MA, USA) according to the instructions as described previously [30]. The ratio of firefly luciferase of each treatment was normalized with Renilla activity.

### 4.3. Cell Viability Assay

The 3-(4,5-dimethylthiazol-2-yl)-2,5-diphenyltetrazolium bromide (MTT) assay was applied to measure the cell viability. BMSCs were isolated from the previous studies were thawed from frozen stock and seeded at 2 × 10^4^ cells/well in 96-well plates overnight, and then exposed to quercetin (Sigma-Aldrich, St. Louis, MO, USA) at different concentrations (0 to 100 nm) at 37 °C in 5% CO_2_ for 3 days. At the end of the treatment, 20 μL of 0.5% MTT was loaded to the culture medium and incubated for an additional 4 h at 37 °C. The supernatant was then removed and the plate was washed with PBS twice. After that, 0.1 mL dimethyl sulfoxide (DMSO) was applied to dissolve the precipitate and the absorbance was measured spectrophotometrically at 570 nm.

### 4.4. Osteogenesis Induction

BMSCs were seeded in a 12-well plate at a density of 1 × 10^5^ cells/well and cultured with αMEM medium. When the cells reached over 80% confluence, the cells were pre-treated with TNFα (10 ng/mL) for 12 h. After that, the culture medium was replaced by a StemProTM Osteogenesis Differentiation Kit (Life Technologies, Carlsbad, CA, USA) together with quercetin (5 and 25 μM, respectively). The culture medium was exchanged twice a week and the BMSCs were kept in 5% CO_2_ at 37 °C. The ALP staining was performed 7 days after induction. The Alizarin Red S (ARS) staining was applied 14 days after osteogenic induction, as described before [31]. The stained patterns were air dried and scanned by Epson Perfection V850 (Seiko Epson, Suwashi, Japan).

### 4.5. RNA Isolation and Real-Time PCR

Total RNA was isolated by using TRIzol reagent (Invitrogen Life Technologies, Carlsbad, CA, USA), as described previously. The obtained mRNA was then reversely transcribed into cDNA by applying PrimeScript RT Master Mix (TaKaRa, Otsu, Japan) as per the manufacturer’s instruction. The real-time PCR was performed on an QuantStudio 7 Flex Real-time PCR system (Applied Biosystems, Foster City, CA, USA) with Power SYBR Green PCR Mix (Life Technologies, Gaithersburg, MD, USA). For result analysis, the 2^−ΔΔCt^ method was used to calculate the fold change of target genes, and GAPDH was used as a reference gene for normalization. The primer sequence is listed in Supplementary Table S1.

### 4.6. Western Blot

Total protein was extracted from whole-cell lysates by pre-chilled RIPA buffer (Sigma-Aldrich, St. Louis, MO, USA) containing a 1% protease inhibitor cocktail (Roche, Basel, Switzerland). Protein concentration was identified by using the BCA method (Pierce Biotechnology, Rockford, IL, USA). Equal amounts of protein (20 μg) were electrophoresed on 10% SDS-PAGE, and then electroblotted to a PVDF membrane. After blocking within 5% skim milk for 1 h at room temperature, the membranes were incubated with primary antibodies including rabbit anti-NF-κB p65 (1:3000, ab16502, Abcam, Cambridge, UK), mouse anti-β-catenin (1:3000, 610,153, BD, Sparks, NV, USA), mouse anti-β-actin (1:3000, Sc-8432, Santa Cruz, CA, USA), and rabbit anti-lamin B (1:3000, CST-12586, Cell signaling technology, Danvers, MA, USA) at 4 °C overnight and washed with Tris-buffered saline with Tween-20 (TBST) 3 times. Horseradish-peroxidase (HRP)-conjugated secondary antibodies (1:3000, Thermo Fisher, Waltham, MA, USA) were used to incubate the membranes at room temperature for 1 h. After washing with TBST 3 times, the SuperSignal™ & Pierce™ ECL chemiluminescence reagent (Pierce Biotechnology, Rockford, IL, USA) was applied to visualize the target proteins on the X-ray file produced from Kodak film developer (Fujifilm, Tokyo, Japan).

### 4.7. Ovariectomized Osteoporotic Model Mice Experiment

All animal operations were permitted by the Animal Experiment Ethics Committee of the Chinese University of Hong Kong (Ref No. 19-276-HMF) and performed in accordance with the Code of Ethics of the World Medical Association. The surgical operation was performed as described previously [32]. A total of 18 14-week-old C57BL/6 mice (female) were acclimated to the laboratory for 3 days prior to the surgery. Then, a Sham-operation was performed on the mice (*n* = 6) or the animals were surgically ovariectomized (*n* = 12) under anesthesia. After a week of recovery, the OVX animals were randomly divided into 2 groups: OVX control group and OVX orally administrated with quercetin (50 mg/kg) twice per week following the dosage suggestions from previous studies [33]. At 4 weeks post treatment, all animals were sacrificed by an overdose of anesthesia and both femurs of the mice were collected and fixed 24 h by 10% neutral formalin solution.

### 4.8. Micro-Computed Tomography Analysis

The femurs were placed in a 15 mm image tube, submerged within 70% ethanol and then subjected into 360° Micro-CT (Scanco Medical, Wangen, Switzerland) using the following parameters: voltage, 70 kV; isotropic resolution, 10.5 μm; reconstruction threshold, 158 mg hydroxyapatite/cm^3^. For the measurement of trabecular bone, the volume of interest (VOI) inside the distal femurs was defined starting from the growth plate and proceeded at 200 continuous sections (10 μm/section) at proximal sides. Three-dimensional (3D) images were reconstructed and the quantitative parameters of bone volume/total volume (BV/TV), bone marrow density (BMD), trabecular thickness (Tb.Th.), trabecular spaces (Tb.Sp.) and trabecular number (Tb.N.) were calculated as previously described [34].

### 4.9. Immunohistochemistry and Histology

The fixed femurs were transferred into 10% formic acid and decalcified for 14 days at room temperature. The decalcified tissue was then embedded inside paraffin and sectioned to a 7 μm thickness for histological analysis. For immunohistochemistry, primary rabbit anti-OCN (1:100, ab93876, Abcam, Waltham, MA, USA) and rabbit anti-OPN (1:200, ab8448, Abcam, USA) antibodies were used to incubate tissue overnight at 4 °C. After that, the tissue was conjugated with HRP-conjugated secondary goat anti-rabbit antibody (1:3000, Thermo Fisher, Waltham, MA, USA) for 1 h at room temperature. The peroxidase-streptavidin substrate kit (DAKO, Carpinteria, CA, USA) was applied for substrate reaction. Additionally, the images were taken by a semi-automatic digitizing image analysis system (OsteoMetrics, Atlanta, GA, USA) [35].

### 4.10. Statistical Analysis

The results are expressed as the mean ± SD by SPSS 23.0 and were illustrated by GraphPad Prism (GraphPad Software, Version 9, La Jolla, San Diego, CA, USA). The unpaired, two-tailed Student’s *t*-test was used to compare the differences of parametric data while the Mann–Whitney U test for non-parametric data. The significance of difference was defined as *p* values < 0.05.

## Data Availability

The data that support the findings of this study are available from the corresponding authors upon reasonable request.

## Supplementary Materials

The following supporting information can be downloaded at: https://www.mdpi.com/article/10.3390/ijms24065965/s1.
