# Supplementary material for: Long Non-Coding RNA Malat1 Increases the Rescuing Effect of Quercetin on TNFα-Impaired Bone Marrow Stem Cell Osteogenesis and Ovariectomy-Induced Osteoporosis
Source: Int J Mol Sci. 2023 Mar 22;24(6):5965. doi: 10.3390/ijms24065965 (PMC10059267; doi:10.3390/ijms24065965)
Supplement: Supplementary file 1 [file ijms-24-05965-s001.zip › ijms-2226310-supplementary.pdf]

Supplementary Table S1. Primers used for construction and Real-time PCR vector assays

Primers for Real-time PCR				
Gene ID	Accession No.	F/R	Sequence	Product size (bp)
mRunx2	NM_001146038	F<br>R	ACACCGTGTCAGCAAAGC<br>GCTCACGTCGCTCATCTTG	99
mAlp	NM_007431	F<br>R	ACTGCGCTCCTTAGGGCT<br>GGCAGCGTCAGATGTTAATTG	104
mOcn	NM_007541	F<br>R	GGTAGTGAACAGACTCCGGC<br>CAAGCAGGGTTAAGCTCACA	96
mH19	NR_130973	F<br>R	CGGTGTGATGGAGAGGACAGAAG<br>CCAGAGAGCAGCAGGCAAGTGTTAG	104
mHotair	NR_047528	F<br>R	CCTTATAAGCTCATCGGAGCA<br>CATTTCTGGGTGGTTCCTTT	92
mDancr	NR_145131	F<br>R	CCTCTCCCGGATGGCTGTAT<br>AGCCATTCAGTCACGGGTTT	162
mMeg3	NR_003633	F<br>R	GGACTTCACGCACAACACGTT<br>GTCCCACGCAGGATTCCA	71
mMalat1	NR_002847	F<br>R	CCTAACGACTAGCATTGGCA<br>GCACTCTTTCCTGGGCTATC	168
mGapdh	NM_001289726	F<br>R	CGTCCCGTAGACAAAATGGT<br>TTGATGGCAACAATCTCCAC	110

“m” stands for “mouse”.

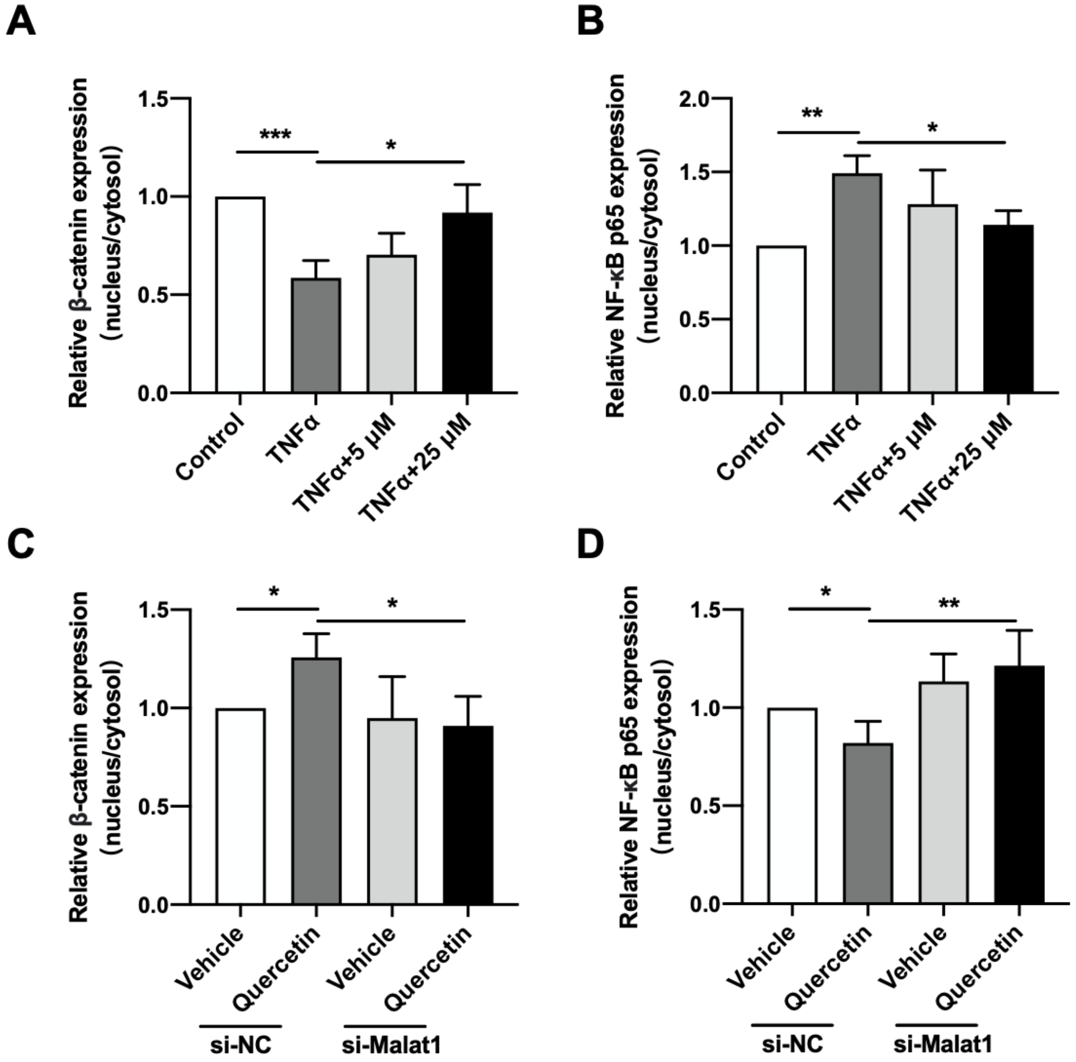

Supplementary Figure S1. A-D. Semi-quantitative analysis of all the Western blot bands in Figure 3A **(A&B)** and Figure 6A **(C&D)** (n=3; *p<0.05, **p<0.01, ***p<0.001).
